# Building Efficient 3D Networks in Polymer Blends by Controlled Capillary Bridging-Induced Particle Agglomeration

**DOI:** 10.1021/polymscitech.5c00056

**Published:** 2025-06-12

**Authors:** Lijun Ye, Ying Tao, Hangchen Cai, Xiaokan Wang, Liping Yang, Yaquan Wang, Yongjin Li

**Affiliations:** † School of Chemical Engineering and Technology, 12605 Tianjin University, 300354 Tianjin, People’s Republic of China; ‡ Key Laboratory of Organosilicon Chemistry and Material Technology of Ministry of Education and Key Laboratory of Organosilicon Material Technology of Zhejiang Province, College of Material, Chemistry and Chemical Engineering, 26494 Hangzhou Normal University, 311121 Hangzhou, People’s Republic of China; § Wankai New Materials Company, Ltd., 314415 Jiaxing, People’s Republic of China

**Keywords:** 3D conductive network, double-percolated structures, cross-linking, capillary force, thermal transport

## Abstract

Multiphase polymer composites offer a versatile platform for constructing efficient 3D conductive networks by regulating filler distribution. However, controlling the spatial distribution and network formation of 2D fillers like boron nitride (BN) flakes in immiscible polymer blends remains a major challenge for achieving efficient thermal conductivity. This work introduces a strategy to regulate the cross-linking degree of low-density polyethylene (LDPE) in poly­(l-lactic acid) (PLLA)/LDPE blends, enabling effective control over BN localization. BN flakes preferentially localize in the LDPE phase, forming double-percolated networks across broad blend compositions. Controlled LDPE cross-linking suppresses domain coalescence and promotes a secondary segregated BN network via capillary bridging-induced agglomeration. This manipulation of domain viscoelasticity enhances 3D filler network formation, increasing the maximum through-plane thermal conductivity of the polymer composites from 2.02 to 2.58 W m^–1^ K^–1^. Our findings offer a facile route for tailoring 3D filler networks in multiphase polymer composites for improved thermal conduction.

## Graphical abstract

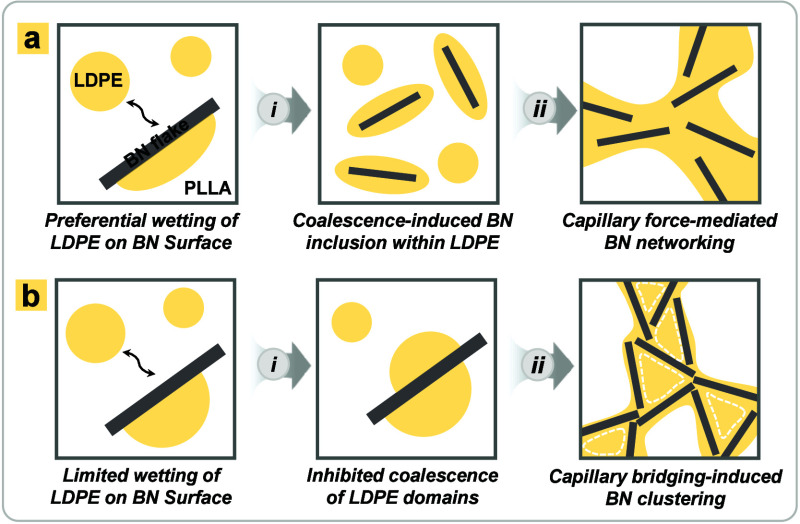

## 1 Introduction

Thermally conductive polymer composites have become promising materials for heat management in advanced electronics, particularly as components become increasingly integrated and miniaturized.^1,2^ To enable efficient thermal transport, the formation of interconnected 3D filler networks in the polymer matrix is crucial.^3,4^ One popular approach to creating polymer composites with high thermal conductivity involves impregnating polymers or polymer precursors into pre-fabricated 3D filler skeletons.^5^ Recently, ice,^6,7^ sugars,^8,9^ and salts^10,11^ have been used as removable templates for constructing 3D skeletons. Alternatively, polymer granules can be used to simultaneously serve as the matrix and structural template, facilitating the formation of segregated filler networks, and making this method applicable to various thermoplastics.^12,13^ In addition, rigid microspheres with sizes comparable to asymmetric fillers can act as building blocks that regulate filler orientation and exfoliation, thus promoting the formation of efficient 3D conductive networks.^14,15^

Double-percolated structures, which enable precise control of filler distribution within one phase or at the interfaces of polymer blends, have demonstrated remarkable success in developing electrically conductive polymer composites with exceptionally low percolation thresholds.^16–19^ However, research on thermally conductive polymer composites incorporating double-percolated structures remains relatively limited.^20–23^ Nanoparticles such as silica dioxides,^24^ carbon black,^25^ and carbon nanotubes (CNTs)^26,27^ have been extensively utilized to construct double-percolated structures in multiphase polymer blends. The spatial distribution of fillers in polymer blends is typically predicted based on the wetting coefficient, which reflects the competitive wetting behaviors of the polymer components at the particle surface.^24,28^ Due to interfacial instability, 1D fillers like CNTs tend to migrate more rapidly across the interface compared to spherical or flaky particles.^28^ In contrast, 2D flaky fillers are more likely to become trapped at the interface.^29^ This inherent tendency may contribute to the limited studies on double-percolated structures involving 2D flaky fillers.^23,30,31^

The interfacial confinement of particles can serve as a physical barrier, preventing the coalescence of polymer domains.^17,32^ For example, CNT networks formed at the interface can stabilize phase morphology by inhibiting domain coalescence, acting similarly to Pickering emulsifiers.^33–35^ This effect is likely to be even more pronounced for 2D flaky particles, whose lateral size may be comparable to or even exceed those of the polymer domains. Additionally, the viscoelastic properties of polymer domains play a crucial role in governing their interactions with dispersed particles, particularly in regulating local wetting behaviors at particle surfaces. These interactions can significantly influence the extent of domain coalescence and the overall phase morphology.^32^

A key question is whether and how domain coalescence in multiphase polymer systems can be controlled by tuning viscoelasticity to facilitate the formation of efficient 3D networks with 2D fillers. Slight cross-linking can reduce the spreading of polymer domains on particle surfaces, thereby helping to regulate domain coalescence.^32^ In turn, these polymer domains may help create a 3D filler network within multiphase polymer blends due to the capillary force-induced particle agglomeration.^36,37^ As a proof of concept, multiphase polymer composites composed of poly­(l-lactic acid) (PLLA), low-density polyethylene (LDPE), and boron nitride (BN) platelets were selected as the model system. LDPE was micro-cross-linked during melt processing using dicumyl peroxide (DCP) as the initiator. The effects of LDPE content and cross-linking density on the structural development and thermal conductivity (*λ*) of PLLA/LDPE/BN composites were systematically investigated. By adjusting the cross-linking density of LDPE, a secondary segregated BN network can be established within the bicontinuous structure of polymer blends.

## 2 Results and Discussion

As a typical 2D filler (Figure 1a), BN flakes are expected to be predominantly trapped at the interface of PLLA/LDPE blends. To investigate the interaction between BN and the polymer components, 10 wt % BN flakes are incorporated into PLLA and LDPE, respectively (Figure 1b,c). Interfacial gaps are observed between the BN flakes and the PLLA matrix, whereas no such gaps are discernible at the LDPE/BN interface. PLLA/LDPE blends with LDPE contents up to 50 wt % exhibit typical droplet-matrix phase morphologies, where the LDPE domains disperse in the PLLA matrix **(** Figure 1d–f and Supporting Information Figure S1a). The size of the LDPE domains is comparable to the lateral size of the BN flakes (∼20 μm). When 50 wt % BN flakes are incorporated into the PLLA/LDPE blends, the LDPE domains are significantly stretched and deformed (Figure 1g–i and Figure S1b). The deformation of LDPE domains is attributable to their preferential wetting and spreading on the BN surface due to the favorable affinity.^38^ Therefore, the LDPE domains may facilitate the formation of 3D BN networks through capillary force-induced agglomeration. This effect becomes even more pronounced when the weight fraction of BN flakes is reduced (Figure 2).

**Figure 1:**
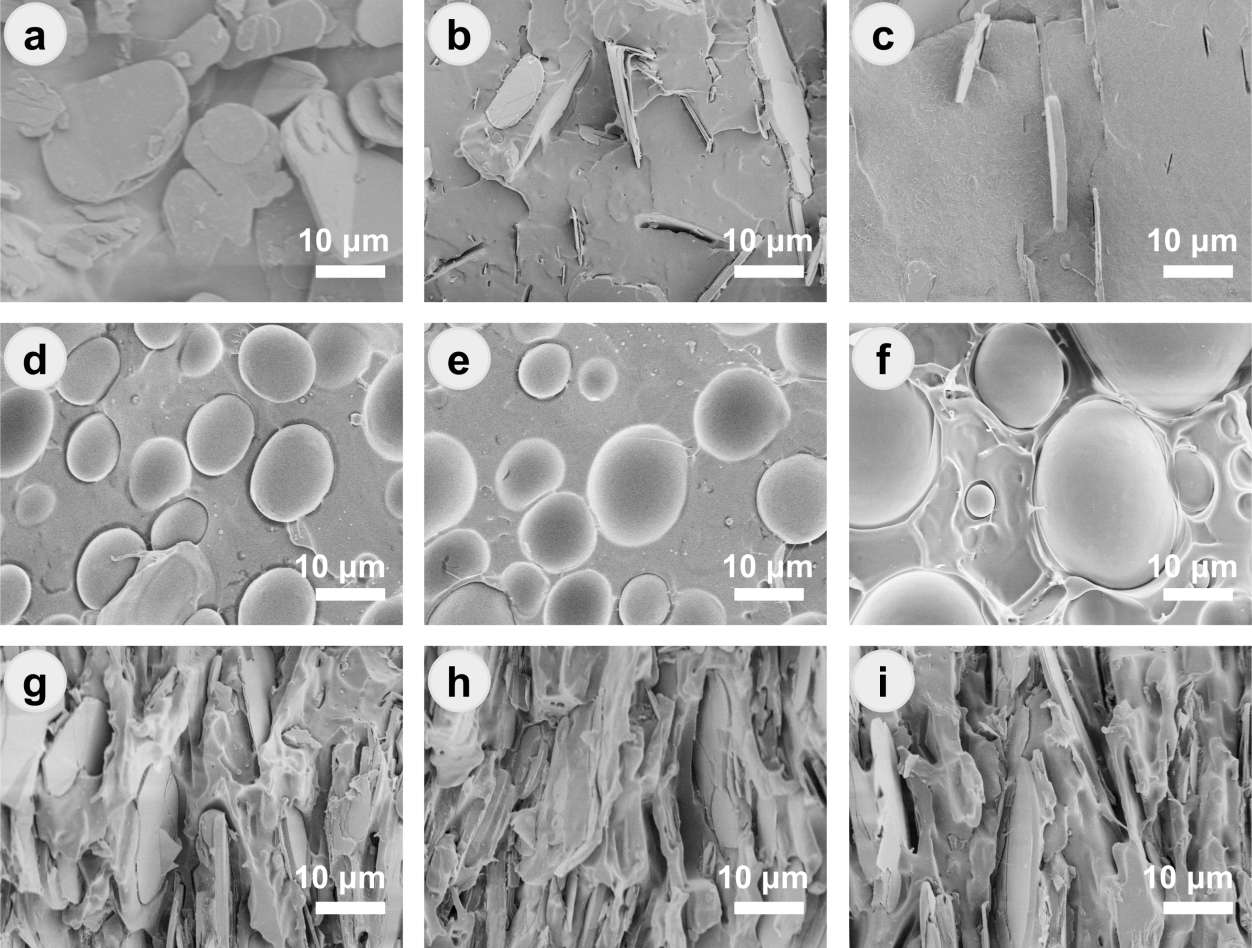
SEM images of (a) BN flakes, (b) PLLA/BN (90/10), (c) LDPE/BN (90/10), (d) PLLA/LDPE (80/20), (e) PLLA/LDPE (70/30), (f) PLLA/LDPE (60/40), (g) PLLA/LDPE/BN (40/10/50), (h) PLLA/LDPE/BN (35/15/50), and (i) PLLA/LDPE/BN (30/20/50).

**Figure 2:**
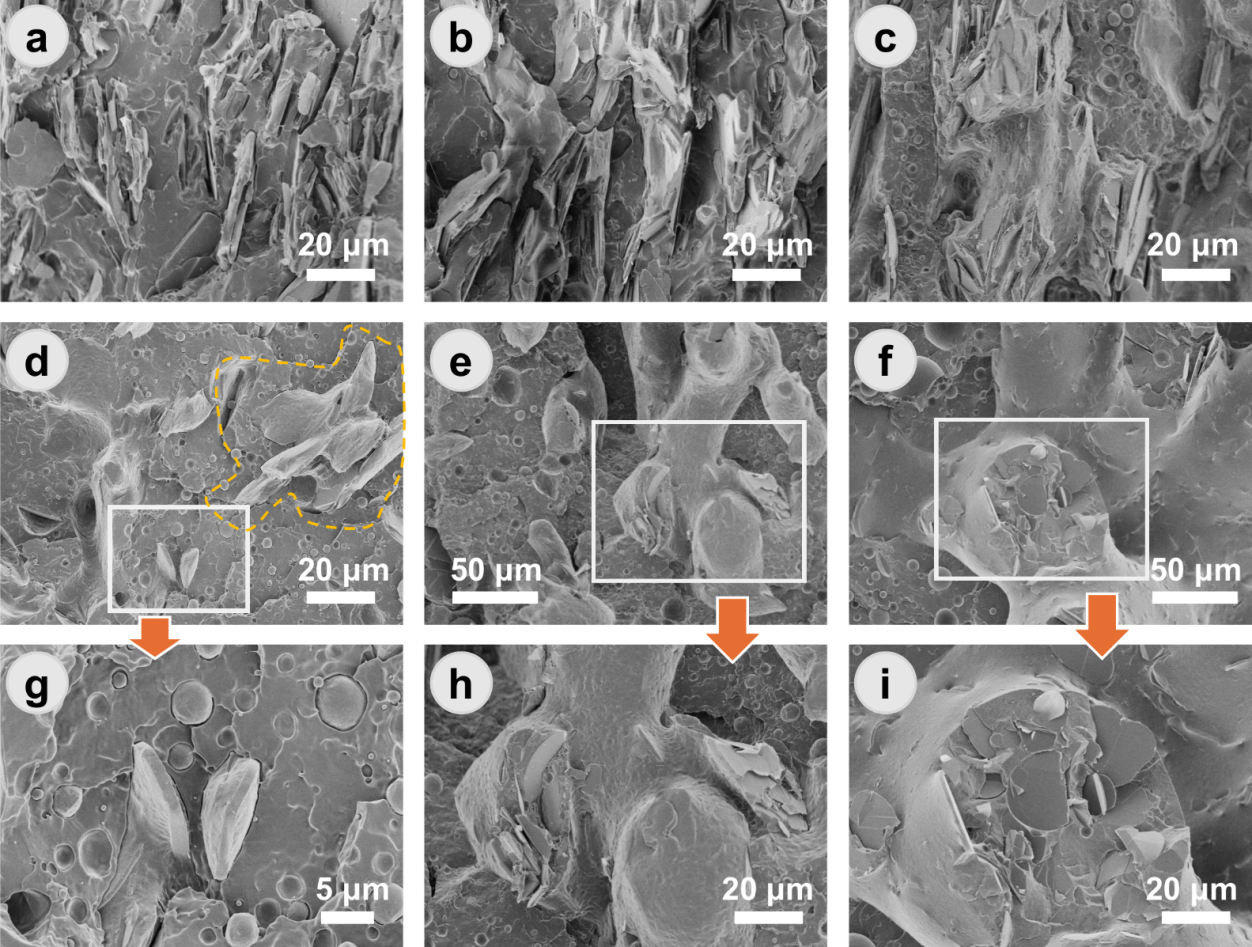
SEM images of (a) PLLA/LDPE/BN (63/7/30), (b) PLLA/LDPE/BN (56/14/30), (c) PLLA/LDPE/BN (49/21/30), (d) PLLA/LDPE/BN (81/9/10), (e) PLLA/LDPE/BN (72/18/10), and (f) PLLA/LDPE/BN (63/27/10) and (g–i) enlarged areas of panels d–f.

Panels a–c of Figure 2 illustrate the evolution of phase morphology in PLLA/LDPE blends containing 30 wt % BN flakes as the LDPE content increases from 10 wt % to 30 wt % within the polymer matrix. The interfacial interactions between BN flakes and LDPE domains, along with the spatial distribution of BN flakes, are clearly visible. At low LDPE content, no discrete domains are observed due to the preferential wetting of the LDPE domains on the surface of the BN flakes (Figure 2a). The high aspect ratio of the BN flakes induces substantial deformation of the LDPE domains. With the increase of LDPE contents, double-percolated structures can be observed where BN flakes are selectively located in the LDPE phase (Figure 2b,c and Figure S2).

To further demonstrate the interfacial interactions between BN flakes and LDPE domains, the weight fraction of BN flakes was further reduced to 10 wt % (Figure 2d–i). In this case, the wetting behavior of LDPE domains on the BN surface can be clearly observed (Figure 2d,g). The “fin-shaped” structures in Figure 2g indicate a strong affinity between the BN flakes and LDPE domains. Capillary force-induced clustering of BN flakes in the PLLA matrix is observed when adding small amounts of LDPE (Figure 2d, outlined by the orange dashed line). As the LDPE content increases, the coalescence of (fin-shaped) LDPE domains and isolated free domains results in the inclusion of BN flakes within the LDPE phase (Figure 2e,f and Figure S3). These observations provide direct evidence for the migration of 2D flakes during dynamic domain coalescence under intensive shear forces during melting compounding.

As mentioned, the location of (nano)­particles in multiphase polymer blends is typically predicted based on wetting coefficients from a thermodynamic viewpoint. The selective distribution of particles is often attributed to competitive wetting-induced migration at the interface (Figure 3a­(i)). When *γ*_BC_ – *γ*_AC_ – *γ*_AB_ *>* 0, particles will migrate into polymer A. Nevertheless, 2D flakes with large lateral dimensions (up to several tens of microns) are expected to be readily trapped at the interface. However, capillary force-induced agglomeration and domain coalescence provide an alternative mechanism for the migration of 2D flakes in multiphase polymer blends (Figure 3a­(ii), as demonstrated by the results in Figure 2. The capillary force-induced particle clustering results in the formation of 3D networks in PLLA/LDPE blends incorporating only 10 wt % BN flakes, with the LDPE content in the polymer matrix reduced to as low as 20 wt % (Figure 3b,c).

**Figure 3:**
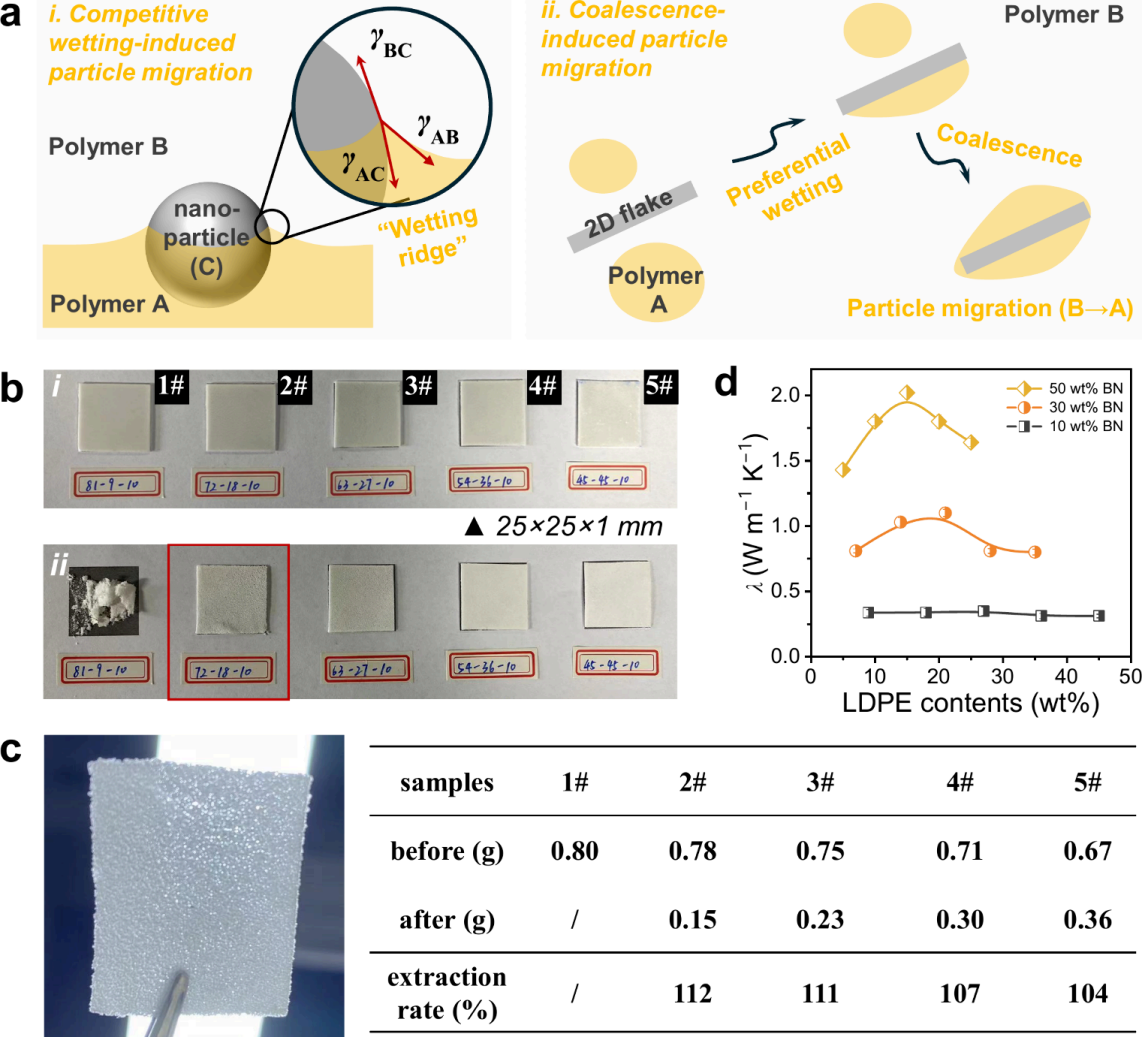
(a) Schematic illustration of (i) competitive wetting and (ii) coalescence-induced particle migration. (b) Optical images of PLLA/LDPE/BN composites (i) before and (ii) after solvent extraction of PLLA: (1#) 81/9/10, (2#) 72/18/10, (3#) 63/27/10, (4#) 54/36/10, and (5#) 45/45/10. (c) Optical image of a free-standing film of 72/18/10 after solvent extraction and calculated PLLA extraction rate of the samples. (d) Thermal conductivity variations of PLLA/LDPE/BN composites upon increasing the LDPE contents.

Figure 3b shows the optical macrographs of PLLA/LDPE blends incorporating 10 wt % BN flakes, with the LDPE content ranging from 10 wt % to 50 wt % in the polymer matrix. To evaluate the network connectivity of BN flakes, all samples were subjected to solvent extraction to remove the PLLA phase (Figure 3b­(ii). The samples with the LDPE content as low as 20 wt % maintain their original shape after solvent extraction (Figure 3c). The PLLA extraction rate was calculated for samples incorporating more than 10 wt % BN flakes. All these samples exhibit a PLLA extraction rate exceeding 100%, which can be attributed to the removal of isolated LDPE/BN domains, particularly at low LDPE contents. In this regard, a reduction in the extraction rate indicates enhanced network connectivity of BN flakes. As the weight fraction of BN flakes increased to 30 wt % and 50 wt %, all samples retained their original shape, exhibiting high PLLA extraction rates (Figure S4 and Table S1).

Figure 3d shows the variations in through-plane thermal conductivity (*λ*) of polymer composites with different LDPE contents (namely, PLB-10, PLB-30, and PLB-50). A rise-and-decline trend in thermal conductivity of the samples with increasing LDPE content is evident across all BN loadings (detailed values are summarized in Table S2). The increase in thermal conductivity is attributed to the preferential wetting of LDPE on the BN surface and the capillary force-induced agglomeration of BN flakes, which facilitates the formation of 3D thermally conductive networks. Domain coalescence could help enhance the network connectivity of BN flakes; however, as the LDPE content increases, the BN flakes will become increasingly “diluted” once they are fully incorporated into the LDPE phase. As a result, the thermal conductivity of the samples begins to decrease due to the reduced effective concentration of BN flakes.

To investigate the potential of LDPE domains as templates for guiding the formation of segregated BN networks by confining BN flakes between them, LDPE was further cross-linked in a controlled manner to adjust its viscoelasticity, which could influence domain coalescence. LDPE was modified through melt blending with varying DCP contents to achieve controlled cross-linking. Panels a and b of Figure 4a show the storage modulus (*G*′) and complex viscosity (|η*|) of LDPE cross-linked with varying DCP contents. Both *G*′ and |η*| increased as the DCP content increased from 0.1 wt % to 0.7 wt %, indicating the increase of the cross-linking degree of LDPE. The cross-linking degree of LDPE, evaluated by the gel content (*C*_g_) and summarized in Table S3, increased from 1.2% (0.1 wt % DCP) to 75.9% (0.7 wt % DCP), consistent with the *G*′ and |η*| changes. Slight cross-linking modifies the wetting behavior of LDPE on the BN surface to suppress domain coalescence. The increased elasticity constrains the formation of the “wetting ridge” typically induced by interfacial tension during the wetting of LDPE domains on BN surface. Meanwhile, the elevated viscous forces counteract the capillary forces at the wetting ridge, thereby slowing the wetting dynamics.^32^ Therefore, the coalescence of LDPE domains was significantly suppressed by BN flakes with a lateral size comparable to the domain size.

**Figure 4:**
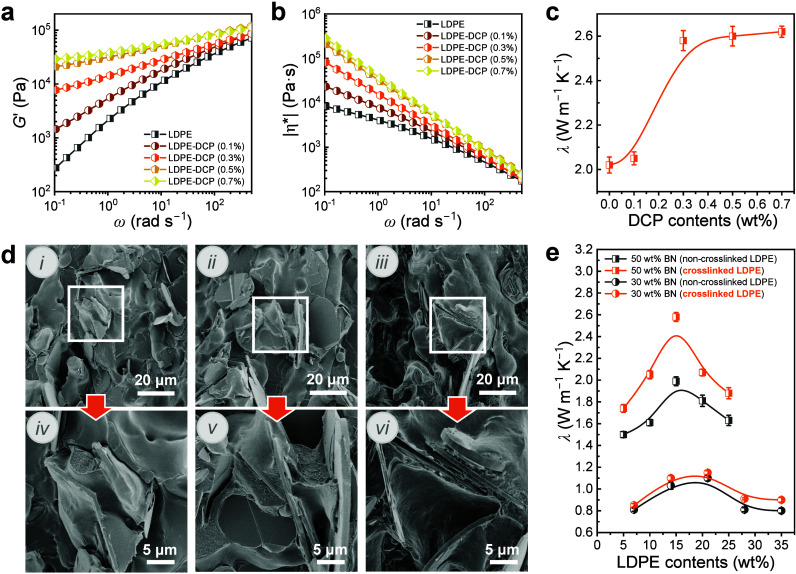
(a) Storage modulus (*G*′) and (b) complex viscosity (|η*|) of LDPE modified with varying DCP contents. (c) Thermal conductivity (λ) variation of PLB-50 samples with a PLLA/LDPE composition of 7/3 as a function of DCP content. (d) SEM images of PLB-10 with PLLA/LDPE compositions of (i) 9/1, (ii) 8/2, and (iii) 7/3. (e) Comparisons of *λ* dependence in PLB-*x* samples as a function of LDPE content.

The thermal conductivity of PLB-50 with a PLLA/LDPE composition of 7/3 was measured with varying LDPE cross-linking degrees (Figure 4c). The through-plane thermal conductivity of the composites increased from 2.02 W m^–1^ K^–1^ for non-cross-linked LDPE to 2.58 W m^–1^ K^–1^ for cross-linked LDPE with 0.3 wt % DCP (*C*_g_ = 56.3%) and stabilized at 2.62 W m^–1^ K^–1^ with 0.7 wt % DCP. Achieving a through-plane thermal conductivity exceeding 2.5 W m^–1^ K^–1^ at a moderate BN content through conventional melt processing is non-trivial, particularly when benchmarked against previously reported values (Figure S5). As previously discussed, slight cross-linking could reduce the spreading of LDPE domains on the BN flakes to suppress their coalescence. In turn, these domains may serve as a template for the formation of secondary segregated BN networks within the LDPE phase. The LDPE cross-linked with 0.3 wt % DCP was used for further investigations.

To investigate the effect of slight LDPE cross-linking on BN network formation, the microstructures of PLB-10 with cross-linked LDPE domains (*C*_g_ = 56.3%) were analyzed (Figure 4d and Figure S6). BN flakes are clearly observed between adjacent LDPE domains with limited coalescence (Figure 4d). The network connectivity was further investigated by solvent extraction to remove the PLLA phase. All the PLB-10 samples maintain their original shape and exhibit high extraction rates exceeding 97% (Figure S7 and Table S4). Thermal conductivity variations of PLB-30 and PLB-50 with slight LDPE cross-linking were evaluated (Figure 4e and Table S5). The thermal conductivity of PPB-50 with cross-linked LDPE domains is consistently higher than that of the non-cross-linked domains across a broad range. These results demonstrate that slight LDPE cross-linking promotes segregated BN networks in double-percolated PLLA/LDPE blends, enhancing heat transfer efficiency across the sample. Notably, slight cross-linking modifies the viscoelasticity of LDPE to effectively regulate the domain coalescence but exerts minimal impact on the mechanical properties of the composites (Figure S8). The impact strength of PLB-50 with 30 wt % LDPE decreased from 10.4 to 8.6 kJ m^–2^, yet remained sufficient for practical applications.

The proposed mechanism underlying the enhanced network formation of BN flakes in PLLA/LDPE blends is illustrated in Scheme 1. As illustrated in Scheme 1a­(i), the preferential wetting and spreading of LDPE domains on BN flakes promote the migration of BN flakes into LDPE domains. Following coalescence, the deformed LDPE domains readily transform into a continuous phase, producing double-percolated structures (Scheme 1a­(ii)). However, with slight cross-linking, the spreading of LDPE domains on BN flakes is restricted, effectively inhibiting their coalescence (Scheme 1b­(i)). These LDPE domains act as templates during capillary bridging-induced agglomeration (Scheme 1b­(ii)), facilitating the formation of a segregated BN network within the LDPE phase. As a result, a well-connected and efficient BN network can be achieved in PLLA/LDPE blends containing slightly cross-linked LDPE domains.

**Scheme 1:**
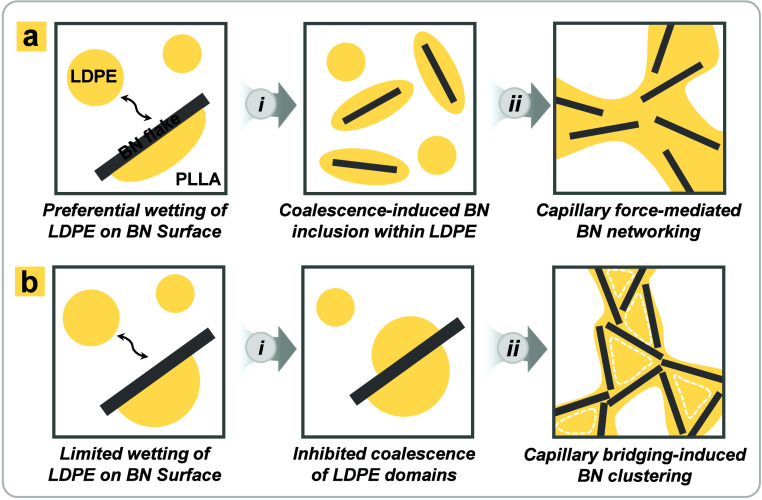
Formation of BN Networks in PLLA/LDPE Blends with (a) Non-cross-linked versus (b) Slightly Cross-Linked LDPE Domains

## 3 Conclusions

In this work, we demonstrate the possibility of constructing efficient 3D BN networks in PLLA/LDPE blends by controlling the spreading of LDPE droplet domains on BN flakes and regulating their coalescence. The preferential wetting and spreading of LDPE domains promote capillary force-driven particle aggregation, forming double-percolated structures after domain coalescence with BN flakes selectively confined in the LDPE phase. Slight LDPE cross-linking will restrict its spreading on BN flakes and suppress domain coalescence. These confined LDPE domains serve as templates for a secondary segregated BN network within the LDPE phase, significantly enhancing heat transport efficiency. The maximum through-plane thermal conductivity of PLB-50 with slight LDPE cross-linking increased from 2.02 W m^–1^ K^–1^ (non-cross-linked LDPE) to 2.58 W m^–1^ K^–1^. This work provides a new approach to achieving efficient 3D conductive networks by tuning viscoelasticity to regulate capillary force-induced particle aggregation and domain coalescence.

## 4 Experimental Section

### 4.1 Materials and sample preparation

PLLA (4032D, *M*_n_ = 8.5 × 10^4^ g/mol, *M*_w_/*M*_n_ = 1.7) was sourced from NatureWorks (USA). LDPE (2426H, *M*_n_ = 1 × 10^4^ g/mol) was acquired from SINOPEC Maoming Petrochemical Co. Ltd. (China). BN flakes with a lateral size of ∼20 μm and a typical thickness of 0.85 μm (Figure S9) were provided by Suzhou Ji’an Napu Material Technology Co. Ltd. (China). Prior to use, all the materials were dried in an oven of 80 °C for 12 h. BN flakes were melt-compounded with PLLA and LDPE by a in a torque rheometer (Haake PolyLab OS, Germany) at 190 °C with a rotation speed of 50 rpm for 10 min. The cross-linking of LDPE was performed by melt-blending with DCP (0.1 wt % to 0.7 wt %) at 190 °C for 10 min. The gel content of LDPE after cross-linking was evaluated after Soxhlet extraction with toluene for 72 h. PLB-*x* composites (where *x* = BN weight fraction) were processed into thin films or disks via hot compression (200 °C, 10 MPa) for SEM characterization and thermal conductivity measurements.

### 4.2 Characterizations

The microstructures of the polymer composites were inspected by a scanning electron microscopy (Hitachi, S4800) with an accelerate voltage of 3 kV. To evaluate the network connectivity, all the samples were subjected to Soxhlet extraction for 72 h to remove the PLLA phase by chloroform. The through-plane thermal diffusivity (*α*) was measured using a Nano Flash apparatus (NETZSCH, LFA 447) on disk samples (2 mm thickness × 12.7 mm diameter). Thermal conductivity (*λ*) was calculated as *λ* = *ρ*·*α*·*C*_*p*_, where *ρ* is mass density and *C*_*p*_ is specific heat capacity. Density (*ρ*) was determined using an electrical densimeter (See Soa Fa, SD 3005) with at least 6 parallel measurements for averaging. *C*_*p*_ values were obtained by DSC (20 °C to 100 °C at 20 °C/min, with 5 min equilibration at 20 °C) using sapphire as standard, with baseline, benchmark, and sample scans performed. Small-amplitude oscillatory shear (SAOS) measurements of cross-linked LDPE were performed using an MCR 301 rheometer (Anton Paar) with parallel-plate geometry (40 mm diameter, 1 mm gap) at 200 °C. Frequency sweeps (500 to 0.1 rad/s) were conducted at 0.5% strain amplitude under air atmosphere.

## Supplementary Material



## Notes

Microstructures (Figures S1–S3, S6); microstructures and etching rates (Figure S4, Table S1); thermal conductivities (Tables S2, S5); gel contents (Table S3); thermal condictivity comparisons (Figure S5); optical images and etching rates (Figure S7, Table S4); impact strengths (Figure S8); size distributions and thicknesses (Figure S9) (DOCX)
