# Supplementary material for: Building Efficient 3D Networks in Polymer Blends by Controlled Capillary Bridging-Induced Particle Agglomeration
Source: Polym Sci Technol. 2025 Jun 12;1(7):632–9. doi: 10.1021/polymscitech.5c00056 (PMC13052687; doi:10.1021/polymscitech.5c00056)
Supplement: Supplementary file 1 [file ps5c00056_si_001.docx]

*Supporting Information*

Building Efficient 3D Networks in Polymer Blends by Controlled Capillary Bridging-Induced Particle Agglomeration

*Lijun Ye,^[[1]](#footnote-1)^*,^a,b^ Ying Tao,^b^ Hangchen Cai, ^b^ Xiaokan Wang,^b^ Liping Yang,^c^ Yaquan Wang,^a^ and Yongjin Li**^,b^

^a)^ School of Chemical Engineering and Technology, Tianjin University, 300354 Tianjin, P. R. China

^b)^ Key Laboratory of Organosilicon Chemistry and Material Technology of Ministry of Education and Key Laboratory of Organosilicon Material Technology of Zhejiang Province, College of Material, Chemistry and Chemical Engineering, Hangzhou Normal University, 311121 Hangzhou, P. R. China

^c)^ Wankai New Materials Co., Ltd, 314415 Jiaxing, P. R. China

**Figure S1**. SEM images of (a) PLLA/LDPE (50/50) and (b) PLLA/LDPE/BN (25/25/50).

**Figure S2**. SEM images of (a) PLLA/LDPE/BN (63/27/30), (b) PLLA/LDPE/BN (56/14/30), (c) PLLA/LDPE/BN (49/21/30), and (d) PLLA/LDPE/BN (42/28/30). All the panels share the same scale bar.

**Figure S3**. SEM images of (a) PLLA/LDPE/BN (54/36/10) and (b) PLLA/LDPE/BN (45/45/10). All the panels share the same scale bar.

**Figure S4**. Optical images of PLLA/LDPE/BN (PLB-30) composite with 30% BN flakes before and after solvent extraction of PLLA.

**Table S1**. Extraction rate of PLLA in PLLA/LDPE/BN (PLB-30) composites with 30 wt% BN flakes.

| PLLA/LDPE/BN | 63/7/30 | 56/14/30 | 49/21/30 | 42/28/30 | 35/35/30 |
| --- | --- | --- | --- | --- | --- |
| original (g) | 0.92 | 0.83 | 0.73 | 0.65 | 0.74 |
| after etching (g) | 033 | 0.34 | 0.36 | 0.37 | 0.46 |
| extraction rate (%) | 102 | 95 | 97 | 98 | 93 |

**Table S2**. Thermal conductivity of PLB-10, PLB-30, and PLB-50 with non-crosslinked LDPE.

| PLB-10 | *λ* (W m^−1^ K^−1^) | PLB-30 | *λ* (W m^−1^ K^−1^) | PLB-50 | *λ* (W m^−1^ K^−1^) |
| --- | --- | --- | --- | --- | --- |
| 81/9/10 | 0.34±0.025 | 63/7/30 | 0.81±0.015 | 45/5/50 | 1.43±0.045 |
| 72/18/10 | 0.34±0.029 | 56/14/30 | 1.03±0.033 | 40/10/50 | 1.80±0.033 |
| 63/27/10 | 0.35±0.033 | 49/21/30 | 1.10±0.027 | 35/15/50 | 2.02±0.046 |
| 54/36/10 | 0.31±0.021 | 42/28/30 | 0.81±0.018 | 30/20/50 | 1.80±0.052 |
| 45/45/10 | 0.31±0.014 | 35/35/30 | 0.80±0.022 | 25/25/50 | 1.64±0.032 |

**Table S3**. Gel contents of LDPE (XPE) modified with different DCP contents.

| Notation | DCP content (wt%) | Gel content (%) |
| --- | --- | --- |
| XPE-0.1 | 0.1 | 1.2 |
| XPE-0.3 | 0.3 | 56.3 |
| XPE-0.5 | 0.5 | 72.5 |
| XPE-0.7 | 0.7 | 75.9 |

**Figure S5**. Comparison of the thermal conductivity achieved in this work with values reported in the literature (refs: rGO-BN/TPU[1], BN/LLDPE/PMMA[2], BN/TPU[3], BN/MWCNTs/AlN/PP[4], BN/PMMA[5], BN/EP[6], h-BN/MEOSs[7], BN/MWCNTs/PVDF[8], BN/CF/Fe_2_O_3_/SR[9], h-BN/AgNPs/ANFs[10]; TPU: thermoplastic polyurethane, SR: silicon rubber, MEOSs: multifunctional epoxyorganosiloxanes, ANFs: armed nanofibers, PP: polypropylene, PMMA: poly(methyl methacrylate), EP: epoxy resin, PVDF: polyvinylidene fluoride).

**Figure S6**. SEM images of PLB-10 with PLLA/LDPE compositions of (a) 6/4, (b) 5/5. Note that the gel content of LDPE is 56.3%.

**Figure S7**. (a) Optical images of PLB-10 before and after solvent extraction. (b) SEM images of PLB-10 with PLLA/LDPE compositions of 7/3.

**Table S4**. Extraction rate of PLLA in PLB-10 with slight LDPE crosslinking.

| PLLA/LDPE/BN | 81/9/10 | 72/18/10 | 63/27/10 | 54/36/10 | 45/45/10 |
| --- | --- | --- | --- | --- | --- |
| original (g) | 0.89 | 0.85 | 0.84 | 0.84 | 0.73 |
| after etching (g) | 0.32 | 0.37 | 0.42 | 0.49 | 0.47 |
| extraction rate (%) | 103 | 97 | 99 | 100 | 100 |

**Table S5**. Thermal conductivity of PLB-30 and PLB-50 samples with non-crosslinked and crosslinked LDPE.

| PLB-30 | *λ* (W m^−1^ K^−1^) | | PLB-50 | *λ* (W m^−1^ K^−1^) | |
| --- | --- | --- | --- | --- | --- |
|  | Non-Crosslinked LDPE | **Crosslinked LDPE** |  | Non-Crosslinked LDPE | **Crosslinked LDPE** |
| 63/7/30 | 0.81±0.015 | **0.85±0.015** | 45/5/50 | 1.43±0.045 | **1.74±0.036** |
| 56/14/30 | 1.03±0.033 | **1.10±0.022** | 40/10/50 | 1.80±0.033 | **2.05±0.038** |
| 49/21/30 | 1.10±0.027 | **1.15±0.017** | 35/15/50 | 2.02±0.046 | **2.58±0.041** |
| 42/28/30 | 0.81±0.018 | **0.91±0.009** | 30/20/50 | 1.80±0.052 | **2.07±0.031** |
| 35/35/30 | 0.80±0.022 | **0.90±0.021** | 25/25/50 | 1.64±0.032 | **1.88±0.051** |

**Figure S8**. Impact strength of PLB-50 with non-crosslinked and crosslinked LDPE.

**Figure S9**. (a) Size distribution and (b) thickness of pristine BN flakes.

**References**

1. Wang, S.; He, H.; Ye, X.; Chen, R.; Li, Q.; Huang, B. Design of rGO-BN hybrids for enhanced thermal management properties of polyurethane composites fabricated by 3D printing. *Compos. Sci. Technol.* **2022**, *227*, 109591.
2. You, F.; Ke, X.; Tang, G.; Yan, X.; Chen, R.; Jiang, X.; Yao, C. Enhanced thermal conductivity in boron nitride incorporated polyethylene/polymethyl methacrylate composites via double percolation structure. *Polym. Compos*. **2024,** *45*, 4550-4560.
3. Bashir, A.; Maqbool, M.; Lv, R.; Usman, A.; Guo, H.; Aftab, W. Surface modified boron nitride towards enhanced thermal and mechanical performance of thermoplastic polyurethane composite. *Compos. B Eng.* **2021**, *218*, 108871.
4. Zhang, Y.; Tang, B.; Liu, Y.; Feng, R.; Song, S.; Xiong, C. Dual-direction high thermal conductivity polymer composites with outstanding electrical insulation and electromagnetic shielding performance. *Polym. Compos*. **2020**, *41*, 1673-1682.
5. Oh, H.; Kim, J. Fabrication of polymethyl methacrylate composites with silanized boron nitride by in-situ polymerization for high thermal conductivity. *Compos. Sci. Technol*. **2019**, *172*, 153-162.
6. Cui, Y.; Bao, D.; Xu, F.; Gao, Y.; Zhang, X.; Geng, H.; Zhou, Y.; Zhu, Y.; Wang, H. Fabrication of EVA connected 3D BN network for enhancing the thermal conductivity of epoxy composites. *Compos. B Eng*. **2021**, *221*, 109203.
7. Yun, H.; Han, C.; Park, J.; Kim, Y. Thermal conductivity and mechanical properties of thermally conductive composites based on multifunctional epoxyorganosiloxanes and hexagonal boron nitride. *Ceram. Int.* **2022**, *48*, 24431.
8. Zhang, P.; Ding, X.; Wang, Y.; Gong, Y.; Zheng, K.; Chen, L. Segregated double network enabled effective electromagnetic shielding composites with extraordinary electrical insulation and thermal conductivity. *Compos. A Appl. Sci. Manuf*. **2019**, *117*, 56.
9. Guo, Y.; Qiu, H.; Ruan, K.; Wang, S.; Zhang, Y.; Gu, J. Flexible and insulating silicone rubber composites with sandwich structure for thermal management and electromagnetic interference shielding. *Compos. Sci. Technol*. **2022,** *219*, 109253.
10. Zhuo, L.; Chen, S.; Xie, F.; Qin, P.; Lu, Z. Toward high thermal conductive aramid nanofiber papers: incorporating hexagonal boron nitride bridged by silver nanoparticles. *Polym. Compos*. **2021**, *42*, 1773.

1. * Corresponding authors.

   Emails: yelij@hznu.edu.cn (L. Y.), yongjin-li@hznu.edu.cn (Y. L.) [↑](#footnote-ref-1)
